# Management of peripheral arterial disease in the context of a multidisciplinary limb program

**DOI:** 10.3389/fcvm.2024.1368655

**Published:** 2024-05-01

**Authors:** Richard F. Neville

**Affiliations:** ^1^ Inova Schar Heart and Vascular, Section of Vascular Surgery, Department of Surgery, Fairfax Inova Medical Center, Falls Church, VA, United States; ^2^ Vascular, Wound and Hyperbaric Medicine Services, Inova Health System, Falls Church, VA, United States

**Keywords:** artery, multidisciplinary, amputation, vascular, CLTI

## Abstract

Peripheral artery disease (PAD) continues to increase in prevalence worldwide due to risk factors such as advanced age, diabetes mellitus, and obesity. Critical limb ischemia (CLTI) is the advanced form of PAD that can result in a lack of healing and limb loss as the most devastating consequence. Patients with PAD, especially CLTI, benefit from multidisciplinary care to optimize outcomes by reducing cardiovascular morbidity and mortality and preventing lower extremity amputation. Collaboration between various specialties allows a focus on problems involved in treating the patient with PAD including prevention, screening, medical care, wound care, infection, and revascularization when needed. Although there is no clear definition or consensus on the structure of the PAD team, certain guidelines are applicable to most clinical scenarios emphasizing “provider champions” in leading a clinical program. A vascular specialist (vascular surgery, interventional radiology, interventional cardiology) and a soft tissue specialist (podiatry, plastic surgery) are the typical “champions,” often involving orthopedics, general surgery, vascular medicine, diabetology/endocrinology, infectious disease, nephrology, and rehabilitation medicine. The team should also include wound nurses, nutritionists, occupational therapists, orthotists, pharmacists, physical therapists, prosthetists, and social workers. This paper presents a brief overview of the structure of the multidisciplinary team with key components and functions of such a team to optimize treatment outcomes for PAD and CLTI.

## Introduction

Peripheral artery disease (PAD) is most commonly due to an atherosclerotic process resulting in reduced lower extremity perfusion as a result of flow reduction from stenosis or occlusion of peripheral arteries. Symptoms of PAD range from asymptomatic disease to intermittent claudication, and, finally, chronic limb-threatening ischemia (CLTI) manifest as rest pain and tissue loss. Physiologic parameters of PAD focus on the ankle brachial index (ABI, <0.9), although other parameters such as the toe brachial index (TBI) have been included in recent publications addressing global standards (1). PAD affects over 10% of adults in the United States with at least 12 million people currently living with this condition, and this number is increasing rapidly (2). Tremendous clinical and economic burdens are associated with PAD including the cost to the health system of treatment and amputation and reduced quality of life (QOL). Amputation has been shown to include costs that double the original cost of the amputation itself (3). This is a significant consideration as over 1.5 million people are living after limb loss, a number that is increasing due to the aging of the population and the global exponential rise in diabetes mellitus.

## Multidisciplinary approach to optimize PAD management

The contemporary management of PAD, especially CLTI, is optimal in the context of a multidisciplinary program. Such a program requires a coordinated effort of physicians, nurses, allied health professionals, and administrators who are dedicated to the cause of treating PAD and saving and maintaining functional limbs. Prompt and appropriate diagnosis followed by a multidisciplinary approach to treatment is critical to successfully treating patients with PAD (4). Programs should incorporate risk reduction with appropriate medical therapy. A review of medical therapy for PAD is beyond the scope of this overview, but familiarity with antiplatelet agents and anticoagulants in the context of the PAD patient is critical. PAD affects the QOL and longevity of patients with significant mortality rates from not only associated major amputation but also coronary disease and stroke, with 5-year mortality approaching 50%. The rise in the prevalence of diabetes mellitus will undoubtedly lead to an increase in lower extremity ulceration limb threat. A multidisciplinary approach leads to enhanced healing, limb preservation, decreased mortality, and increased patient satisfaction through a decrease in the morbidity and mortality associated with PAD/CLTI.

Unfortunately, there continues to disparity of care based on race, socioeconomic status, and geography (5, 6). A limb preservation program would standardize care for the patients with CLTI mitigating some of the factors that cause disparity in the care. Of those patients with limb-threatening ischemia, 25% undergo primary amputation, 25% receive medical therapy, and 50% undergo attempted revascularization. Goodney et al. (7) have reported that 46% of patients undergo major amputation without a diagnostic arteriogram despite evidence that diagnostic arteriography is a predictive factor for limb preservation. There are data to support the fact that amputation is prevented by an increasing incidence of revascularization whether endovascular or open surgical bypass and that amputation rates are lower in centers with higher volumes of revascularization procedures (8). An important factor to address these issues in such a program is communication. Several medical disciplines can assume leadership roles for such a program in each community. Leadership is based on the passion for the type of patients managed shown by the participants as much as specific medical training. It is important to draw on the expertise and passion available in the local medical community to construct a limb preservation team.

## Components of a limb program

An appropriate staff is the critical component of a multidisciplinary program. The team must share a dedication and passion for the goals as outlined above and create and maintain open lines of communication. The staff includes the physician team, administrative support, physician extenders, nursing team, and secretarial support. A medical director who has the authority and initiative to bring together other team members should lead the physician team. The background of the medical director can vary but is most often drawn from vascular surgery, podiatry, or plastic surgery. It is important to have representatives from both the vascular and soft tissue perspectives in leadership roles, and therefore co-director positions can be created. Although there is not only one model regarding the multidisciplinary PAD team, certain applicable guidelines begin with the importance of “provider champions” in leading a clinical program. A vascular specialist (vascular surgery, interventional radiology, interventional cardiology) and a soft tissue specialist (podiatry, plastic surgery) are the typical “champions,” often involving orthopedics, general surgery, vascular medicine, diabetology/endocrinology, infectious disease, nephrology, and rehabilitation medicine. The team should also include wound nurses, nutritionists, occupational therapists, orthotists, pharmacists, physical therapists, prosthetists, and social workers.

There should also be an administrator dedicated to the program with time available to guide the project on a daily basis. The administrative director can be either a physician (medical director) or someone trained in medical administration. The administrator should know the clinical and business aspects of the program and be facile with interaction among the hospital, physicians, staff, and local community. Physician extenders such as nurse practitioners or physician assistants play a crucial role in the success of the program. These practitioners initiate the medical evaluation of each patient and coordinate the complex care required for an optimal outcome. These providers can often run clinics independently with supervision from treating physicians. This includes wound care, preoperative preparation, and prescription of medications used in the treatment plan. The physician extenders must work closely with trained wound nurses, medical assistants, and secretaries. The wound nurses or technicians are the focus of the clinic setting. These specialized personnel perform much of the wound care and dressing changes and important patient education required to involve the patient in their own care. Coordination of visiting nurse visits is also coordinated by the wound nurses. The ideal complement of such personnel to optimize patient flow is approximately three physician extenders/wound nurses for each doctor involved in the clinic setting. Staff support should also include a receptionist and medical assistants. The medical assistants aid in moving patients through the clinic and completing initial demographic and historical data in the electronic medical records and may help in the removal and application of dressings under the direction of the wound nurse or physician. Ideally, case managers and prosthetists form part of the program. Case managers assist in the volume of work involving rehabilitation centers and insurance issues, which patients often require, while a prosthetist with familiarity with the patients and direct communication with the limb team greatly enhances the functional results of the patients.

A dedicated, identifiable outpatient space is important. The space should be accessible to those patients who have mobility problems. The outpatient space should be in proximity or connected to the hospital, as limb patients require frequent hospital services. The space for exam rooms is also important, as patient flow is important to program viability and patient satisfaction. Ideally, six rooms for every 30 scheduled patients would allow for the optimization of the physician and physician extender team. A designated inpatient hospital ward for limb patients when they are admitted is also preferred. The nursing staff on an identifiable ward should be familiar with the medical issues surrounding the PAD patients. Wound dressings and other medical materials can be centrally located in proximity to the admitted patients. The identifiable limb ward fosters the team approach and continuity of care from outpatient to inpatient setting and back to outpatient wound or rehabilitation centers as needed.

## Non-invasive vascular laboratory

A non-invasive, diagnostic vascular laboratory is critical to the management of PAD and requires appropriate space and equipment. These vascular laboratories are used routinely in the care of the PAD patient both for initial diagnosis, follow-up care, and the decision for invasive therapy. The vascular laboratory should perform the standard tests including ABI, segmental waveforms and pressures, post-volume recordings, digital pressures, and duplex ultrasound imaging. Diabetes is associated with increased calcification of the arterial media, often rendering the tibial arteries incompressible by the blood pressure cuff and a falsely elevated ABI. The TBI is important for the assessment of PAD in diabetic patients and digital pressures to determine healing potential. Other modalities can assist in the assessment of tissue perfusion and healing ability including transcutaneous oxygen measurement and skin perfusion pressure. Transcutaneous oxygen values are measured in the area of the wound and usually at the calf, ankle, or foot with an absolute value of >20–30 mmHg consistent with healing supported by a chest wall index of >0.4. The chest wall index compares the value in proximity to the wound to the value taken on the chest wall, which is considered a normative value. Measuring skin perfusion pressures utilizes a Doppler shift effect in laser light reflected from the capillary flow with the pressure measured at which the blood flow first returns to the capillary bed of the skin envelope. A skin perfusion pressure of >30 mmHg belies CLTI while predictive of healing. Hyperbaric oxygen (HBO) therapy can serve a certain segment of the threatened limb population and is of particular value in patients with irradiation-induced ulcers or diabetic foot ulceration. HBO may be useful if an increase in tissue oxygenation can be demonstrated when the patient is given supplemental oxygen (9). It requires a facility with dedicated staff that recognizes the associated medical risks.

## Arterial imaging in the management of PAD

When intervention is deemed necessary by patient history, exam, and vascular lab studies, then arterial imaging is required to plan the appropriate revascularization, including CT angiography (CTA), MR angiography (MRA), and catheter-based arteriography. CTA allows 3D reconstruction while demonstrating calcium and the calcium load in the arterial tree. Unfortunately, CTA involves contrast with the timing of the injected bolus critical to image quality. Heavily calcified arteries can also impact image quality. MRA is a flow-dependent analysis, and while non-invasive without the need for standard contrast, the best images are obtained with the administration of gadolinium, which is problematic in patients with renal insufficiency due to the chance of nephrogenic systemic fibrosis. Additionally, MRA images can overestimate the degree of stenosis due to signal dropout, and certain patients cannot tolerate the time required to acquire the images in the enclosed space. Catheter-based arteriography continues to play an important role in diagnostic imaging for limb preservation, especially in terms of the precise definition of distal tibial disease. A primary intervention can be performed at the time of the initial arteriogram; however, obtaining quality images of the lower leg and foot with subsequent planning of complex revascularization procedures is often appropriate. This approach allows collaboration and communication among providers of varying skills and experience in the different modes of revascularization.

## Revascularization in the management of PAD

Revascularization to treat PAD is performed to alleviate the symptoms of disabling claudication, enhance wound healing, and prevent amputation. Claudication is rarely a limb-threatening situation, and conservative therapy should always be considered with exercise, tobacco cessation, and the possible addition of cilostazol. The role of endovascular therapy in treating PAD has dramatically increased with many centers advocating an “endovascular-first approach.” This has changed the landscape of lower extremity revascularization for PAD. Recently, two prospective, randomized clinical trials, BEST-CLI and BASIL-2, have published results comparing bypass and endovascular revascularization (10, 11). While both trials emphasized the benefits of revascularization for PAD/CLTI, the difference between trials in choice of primary endpoint led to different interpretations of conclusions. The BEST-CLI trial chose major adverse limb events (MALE) with mortality and concluded that bypass with a saphenous vein was superior to an “endovascular-first” approach. The BASIL-2 trial chose amputation-free survival (AFS) incorporating the primary endpoints of amputation and survival. This trial indicated that endovascular therapy may be preferred based on a survival difference between groups without a difference in amputation rates. Both trials demonstrate that trial surgical bypass and endovascular intervention can be effective modes of revascularization and should be offered in a limb program. An important observation was the persistently high mortality rate of patients with symptomatic PAD/CLTI. This is an area that must be addressed.

Outpatient revascularization is becoming more common and accepted by patients, providers, and insurance carriers. PAD programs often include space for an outpatient office-based lab (OBL) or ambulatory surgery center (ASC). However, procedures performed in these settings must satisfy appropriate indications and diagnostic evaluations prior to PAD intervention. As the number of procedures performed in the OBL setting continues to increase, the need for oversight of outcomes is critical.

## Revascularization techniques

Revascularization in the context of a PAD program focuses on rest pain and tissue loss as primary indications for intervention. This program requires options for both endovascular therapy and open surgical bypass. Patients requiring revascularization can be complex with distal occlusive disease, a lack of autogenous conduit, concomitant infections, and prior failed attempts of revascularization. In terms of endovascular revascularization, the entire range of therapies should be available to treat the patient appropriately; however, a predominance of patients can be treated with standard balloon angioplasty and stent deployment. Atherectomy has a select role, but an increasing role, in certain patients based on the experience of the operator and venue (12). There has been an increased utilization of aggressive endovascular techniques such as extensive tibial angioplasty with crossing of chronic total occlusions and distal pedal loop revascularization (13). Despite controversial data regarding patient mortality (14), the use of drug elution technology will almost certainly have an increasing impact in the future on patients in need of revascularization for limb preservation.

**Figure 1:**
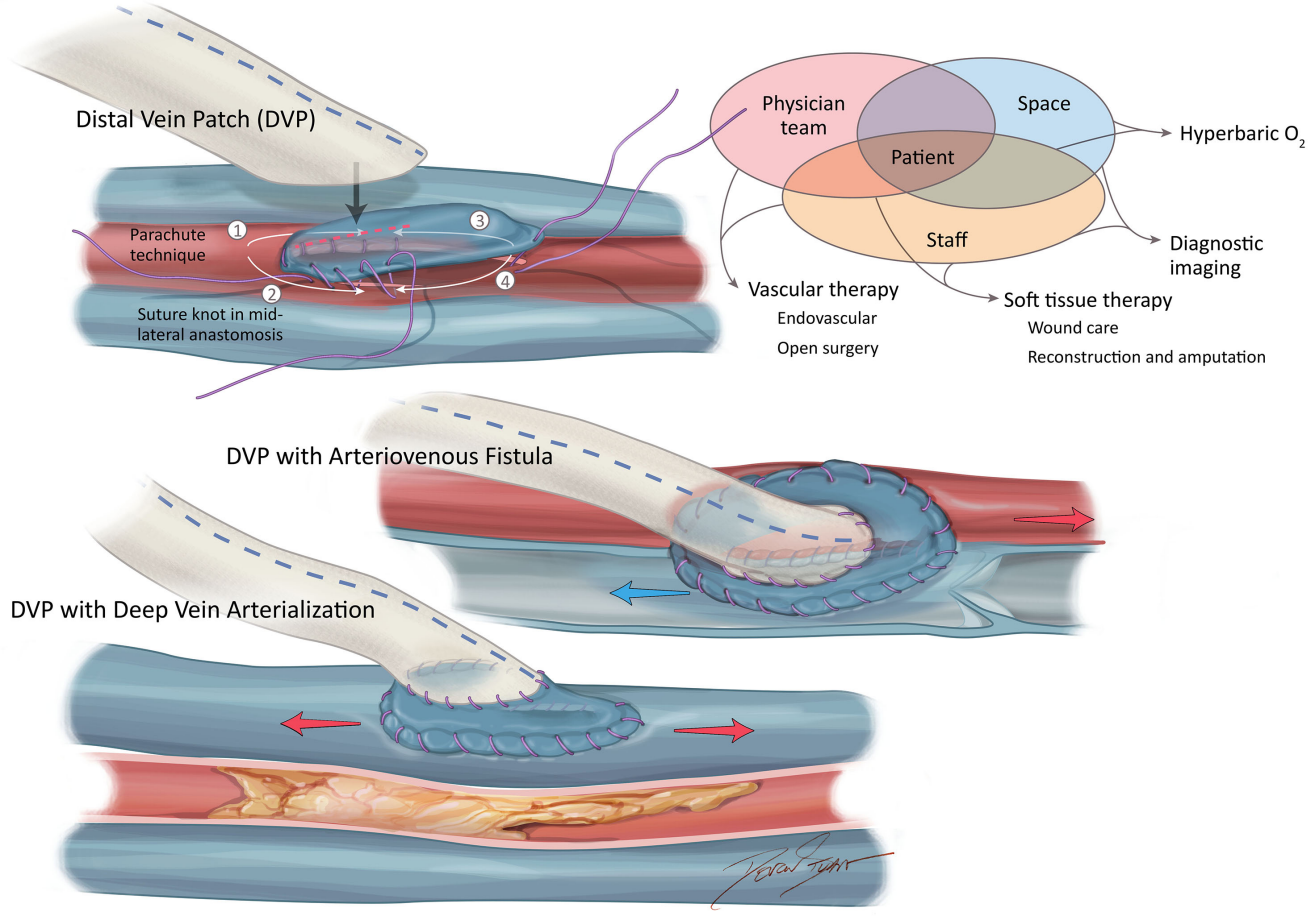
Advanced bypass options for limb preservation.

There is a subset of patients in whom bypass should be considered for optimal revascularization to enhance healing and limb preservation. Hybrid operating room technology has become essential for the complex procedures often required for the revascularization of more advanced PAD. These patients include those presenting with large volume tissue loss (>2 cm), patients with several prior attempts at endovascular therapy, and patients in whom angiosome revascularization may be important with the target artery best perfused with a bypass (15). Development in bypass technique includes heparin-bonded prosthetic conduits with a venous adjunct at the distal anastomosis, the addition of an arteriovenous fistula at the distal anastomosis, and surgical deep venous arterialization with utilization when there is a lack of autogenous conduit, and, in the presence of poor arterial runoff (16–21) (Figure 1). Many bypasses are now performed following endovascular failure and lack of venous conduit. Furthermore, this is complicated by the paucity of suitable distal arterial targets (the “desert foot”) requiring a more distal target than would have previously been required following endovascular therapy.

## Wound care and soft tissue reconstruction

In a PAD program, wound care and soft tissue reconstruction are equally important to revascularization. The majority of leg ulcers can be effectively managed with non-surgical debridement, typically in the form of dressing changes or wound ointments that encourage tissue healing. Whether performed in the operating room or clinic, the goal of debridement is to remove all non-viable, infected tissue and reach bleeding tissue or viable fat, tendons, or fascia. Consideration should be given to the delayed wound closure and the vacuum-assisted closure device (VAC) or other biologic wound care adjuncts. The VAC is a negative pressure wound therapy that enhances granulation tissue ingrowth through reduction of edema and removal of proteases (22). VAC therapy can be discontinued when the wound is small enough to close with simpler less expensive dressing changes or can be closed primarily or with a skin graft. Ideally, wound care should be performed under the auspices of wound care protocols, which can stabilize the uniformity of the procedures and optimize results in a limb preservation program as carried out by the medical and ancillary staff.

The PAD program should include the ability to perform advanced soft tissue flaps when primary wound care is insufficient. Three types of flaps are employed in the lower extremities: local random-pattern flaps, local pedicle flaps, and free tissue transfers. All soft tissue flaps are based on axial vessels, typically branches of arteries supplying the angiosomes to maintain perfusion to the tissue of the flap in question. The use of VAC therapy and other flaps has led to a decline in the use of microsurgical free flaps in the lower extremities. However, there are certain wounds for which free flaps may still be useful, such as large defects and wounds characterized by significant bone exposure.

## Amputation

Although the goal of a PAD program is limb preservation, primary amputation may be required in varying clinical scenarios, such as lack of tissue for reconstruction, sepsis, non-ambulatory status, and/or being demented and unable to cooperate with rehabilitation. The goal of PAD management should be to maximize the functional length of a biomechanically sound limb. Proper limb biomechanics is the key to successful amputation. To optimize biomechanics, anatomy must be appreciated, and the procedure performed with technical care. Viable tissue should be maximized especially along the plantar surface of the foot as this tissue is often used for forefoot reconstruction. Skeletal stability is also important. The patient should not be left with any pressure points or potential areas of decubitus ulceration after amputation. Several amputations can be considered to obtain an optimal result. Minor and below-ankle amputations include digital (ray) amputation, transmetatarsal amputation, and midfoot (Lisfranc), hindfoot (Chopart), and less commonly ankle amputation (Symes). The goal of these procedures is to maximize the length and ambulatory status. Major or above ankle amputations are classified as above- and below-knee. All amputations can be technically challenging to obtain long-term function, but the below-knee amputation must be done especially carefully to design a well-constructed posterior flap with maximal length from the tubercle of the tibia. Tenodesis (the anchoring or fixation of a tendon to a bone) to the tibia helps maintain function and diminish deformity postoperatively. Advances in prosthetic technology have greatly contributed to maintaining ambulatory function after a well-done major amputation. Peer support groups can also help assist the amputee in a successful recovery.

## Education and research

A limb preservation program should also include a patient support group, as there is a significant emotional component to limb loss or living with a foot ulcer. A patient support group can be very important both preoperatively and postoperatively in helping patients deal with the burden of their clinical situation by maintaining functionality and a positive outlook. Education can be incorporated into the program in the form of seminars for patients and referring physicians. Education is critical to achieving the goal of limb preservation through the reduction of amputation and enhancement of the QOL of patients. The limb program also provides a format for multidisciplinary research that is increasingly important to the healthcare system. Both education and research are a reminder of the importance of a patient-centered approach when initiating and maintaining a multidisciplinary limb preservation program.
